# Implementing an Antimicrobial Stewardship Program in an Oncology Center in Lima, Peru: A Model for Low- and Middle-Income Countries

**DOI:** 10.1093/ofid/ofae402

**Published:** 2024-07-12

**Authors:** Carlos Seas, Pedro Legua, Brian Delfin, Karol Villavicencio, Angie Palomino, Paola Montenegro, Ivan Aguilar, Yenka La Rosa, Maribel Robles, Frank Young

**Affiliations:** Clinica Oncosalud, AUNA, Lima, Peru; Clinica Oncosalud, AUNA, Lima, Peru; Clinica Oncosalud, AUNA, Lima, Peru; Clinica Oncosalud, AUNA, Lima, Peru; Clinica Oncosalud, AUNA, Lima, Peru; Clinica Oncosalud, AUNA, Lima, Peru; Clinica Oncosalud, AUNA, Lima, Peru; Clinica Oncosalud, AUNA, Lima, Peru; Clinica Oncosalud, AUNA, Lima, Peru; Clinica Oncosalud, AUNA, Lima, Peru

**Keywords:** antimicrobial stewardship, antimicrobial use, antimicrobials, oncology, surgical prophylaxis

## Abstract

Our center launched the first antimicrobial stewardship program in Peru. From 2016 to 2023, the proportion of antimicrobial prescriptions audited increased from 60% to 95%, and 65% to 95% of recommendations were accepted. Vancomycin and meropenem use dropped by 95% and 84%, respectively. The proportion of recommendations for surgical prophylaxis exceeded 90%.

## Graphical Abstract

**Graphical Abstract:**
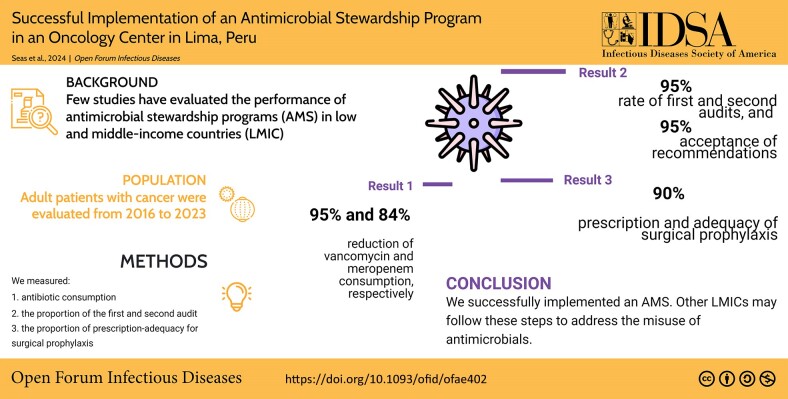
https://tidbitapp.io/tidbits/succesful-implementation-of-an-antimicrobal-stewardship-program-in-an-oncology-center-in-lima-peru-lesson-for-low-and-middle-income-countries-ce8d7bef-68d0-4fc6-8d1e-d9c2aa0920ea

## Teaser

Our center launched the first antimicrobial stewardship program in Peru. Significant achievements included increased audits for antimicrobial prescriptions, particularly for vancomycin and meropenem, and a proportion of recommendations for surgical prophylaxis exceeding 90%.

In 2016, our center became the first institution in Peru's private healthcare sector to implement an antimicrobial stewardship program (AMS). The performance and effects of AMS programs in low- and middle-income countries (LMICs) have not been extensively studied [1], and few studies have focused on patients with cancer [2–4]. In response to the urgent global need to combat antimicrobial misuse [5, 6] and the worldwide rise in antimicrobial resistance, particularly in LMICs [7], we implemented an ambitious AMS program. However, we faced unique challenges: 1 significant challenge was that most of our medical staff worked part-time in the public sector, where they had adopted antimicrobial prescription practices that deviated from the regimens recommended in our center according to our microbiological profile. We therefore developed and implemented a comprehensive intervention strategy designed to foster acceptance of our recommended antimicrobial regimens, optimize antimicrobial consumption, and streamline the use of antimicrobials for surgical prophylaxis. Since 2016, our program has been pivotal in changing prescription behaviors and promoting the rational use of antimicrobials. Moreover, the data we generate are shared with policymakers to help improve national health care. In this report, we present our interventions and results from 2016 to 2023.

## METHODS

Clínica Oncosalud, the largest private oncology institution in Peru, has comprehensive facilities, including 77 admission beds; a 24-hour emergency room; a 6-bed intensive care unit; a laboratory fully equipped for hematological, biochemical, and microbiological testing; a radiology diagnostic unit with computed tomography and magnetic resonance imaging capabilities; a gastroenterology unit; and a surgical unit. A multifaceted AMS program was implemented in 2016 as a result of a 2015 review of data that revealed unnecessarily high consumption of vancomycin and meropenem, alongside inadequate surgical prophylaxis practices. The data for this report were obtained prospectively from 2016 to 2023. The program followed the recommendations of the World Health Organization (WHO) for implementing an AMS program in LMICs, a WHO practical toolkit, and its core components are described in the following sections [8].

### Multidisciplinary Collaboration

The AMS program fosters cooperation among oncologists, surgeons, intensive care unit staff, emergency physicians, infectious disease specialists, microbiologists, epidemiologists, nurses, and pharmacists. Members of each specialty collaborated in designing a comprehensive collaboration plan.

### Documentation of Baseline Microbiological Information

The pathogens isolated in bloodstream, respiratory tract, and urinary tract were reviewed comprehensively. Data were tabulated according to the location of pathogen isolation, age and gender of patient, and type of tumor using the WHONET software. The information was discussed by the AMS team, which included an infectious disease specialist physician, a microbiologist, a pharmacist, and 2 nurses; it was then published every 6 months.

### Local Guidelines and Protocols

We designed guidelines for treating infections and for surgical prophylaxis according to local microbiological data. These guidelines were shared across all specialties through the center's electronic software, which ensured accessibility for all involved personnel.

### Education and Training

Educational materials, developed for review by all health care personnel, included recorded videos, lectures, and printed materials featuring crucial messages about the importance of proper antimicrobial use and the consequences of misuse. Training sessions were scheduled throughout the year to ensure awareness of policies and adherence to best practices.

### Antimicrobial Prescription Audit and Evaluation of Antimicrobial Consumption

In a robust process led by a physician–infectious disease specialist, health care personnel reviewed prescriptions of antimicrobials with high consumption rates (particularly vancomycin and meropenem, which are often misused, as well as ceftriaxone, ciprofloxacin, and voriconazole). This prospective audit was conducted twice for each patient: within 24 hours of the prescription and 72 hours later. The percentages of consultations for both audits and the number of recommendations accepted were tabulated. Immediate feedback was provided to prescribers after each audit. To evaluate antimicrobial consumption, we calculated the defined daily dose according to 100 patient-days (DDD per 100 pd), as recommended by the WHO [8]. In addition, we calculated percentages of antimicrobials among all drugs prescribed and recorded the adequacy of antimicrobial use as prophylaxis for mastectomies and prostatectomies. Our target for both prophylaxis indicators was 90%.

### Pharmacy Interventions

The pharmacy issued an alert whenever a restricted antimicrobial was prescribed. We documented those alerts, as well as each time the electronic record indicated that the medication had been used, for 7 days. This enabled real-time monitoring of antimicrobial prescribing.

### Continuous Quality Improvement

The AMS team convened regularly to assess quality performance indicators. In these meetings, members of each discipline reviewed the data and devised strategies for enhancing quality indicators.

## RESULTS

**Figure 1.**
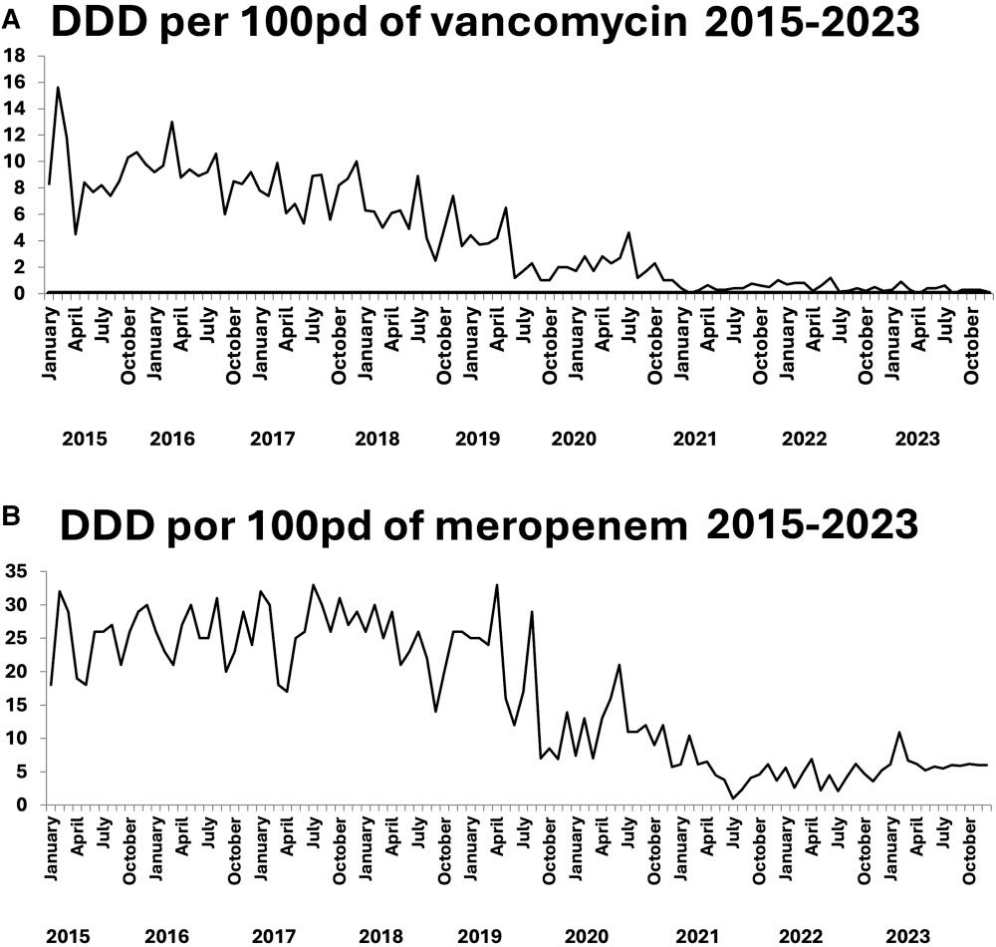
*A*, Marked reduction in the defined daily dose (DDD) per 100 patient-days of vancomycin during the study period. *B*, Marked reduction in the DDD of meropenem per 100 patient-days during the study period.

**Figure 2.**
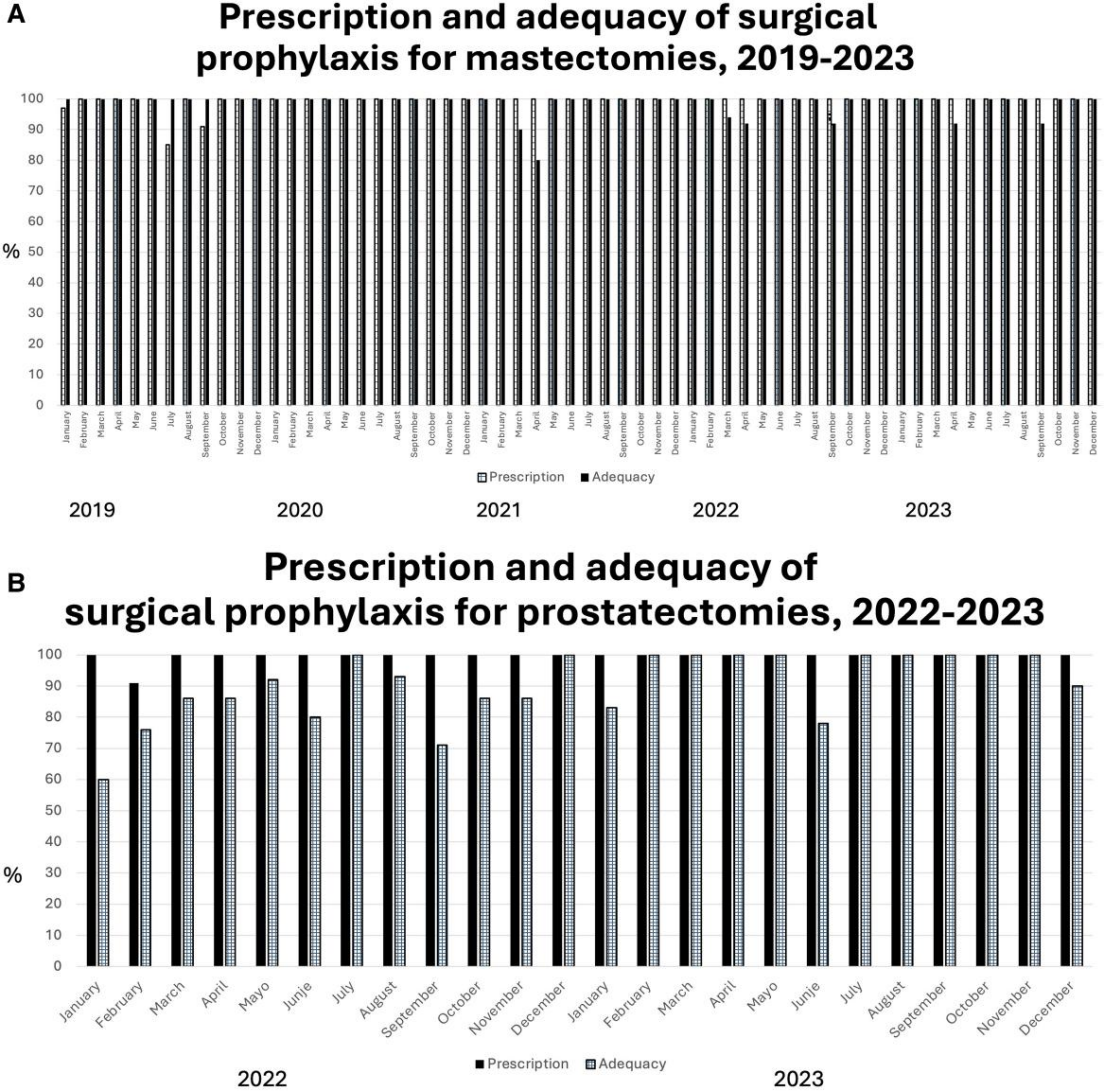
*A*, The changes in the proportion of antimicrobial prescriptions and in the adequacy of antimicrobials as surgical prophylaxis for mastectomies during the study period. These proportions exceeded the 90% prespecified target. *B*, The changes in the proportion of antimicrobial prescriptions and in the adequacy of surgical prophylaxis for prostatectomies during the study period. These values are close to the 90% prespecified target.

The median monthly number of first audits during the study period was 130 (range, 88–188). The proportion of antimicrobial prescriptions that were audited once or twice increased from 60% in 2015 to 95% in 2023; 65%–95% of recommendations in these audits were accepted. Over the 8-year study period, vancomycin consumption decreased 95% (from 9.3 to 0.5 DDD per 100 pd), which coincided with the near absence of methicillin-resistant *Staphylococcus aureus* (MRSA; Figure 1*A*). Moreover, meropenem consumption decreased by 84% (from 25 to 4 per DDD per 100 pd; Figure 1*B*). Our center's microbiological profile did not change substantially during the study period (Supplementary Supplement 1). The 5 most common bacteria in bloodstream infections were *Escherichia coli* (37% of infections; 52% of cultured organisms produced extended-spectrum beta-lactamases [ESBLs]), *Klebsiella pneumoniae* (17% of infections; 60% of cultured organisms produced ESBLs), *S aureus* (4% of infections; no MRSA was isolated), *Pseudomonas aeruginosa* (4% of infections), and *Enterococcus faecalis* (4% of infections). The mean number of bacterial isolates per year was 900. Since 2019, the overall proportion and adequacy of prescriptions of antimicrobials for prophylaxis in mastectomies and prostatectomies have consistently exceeded 90% (Figure 2*A* and *B*). Meanwhile, the rate of in-hospital all-cause mortality remained stable at 3%.

## DISCUSSION

Our findings highlight the feasibility of implementing a multifaceted intervention to enhance antimicrobial use within oncology centers in resource-limited settings. Crucial to achieving these suitable quality indicators is the dedication of personnel and the political commitment of institutional leaders. The significant achievements—notable increases in infectious disease consultations and in acceptance of recommendations, the reduction in the prescription of 2 critically misused antibiotics, and the improvement in prescription and adequacy of surgical prophylaxis—underscore the success of our AMS program, which can serve as a model for other institutions aiming to implement similar programs.

Patients in oncology centers are particularly vulnerable to the consequences of inappropriate use of antimicrobials. Many patients are so ill that physicians initiate antimicrobial treatment even in the absence of symptoms or signs of infection [9]. Our center's protocol was no exception to this practice; most of the patients in 2015 were empirically administered vancomycin and meropenem under the assumption that MRSA- and ESBL-producing strains of Enterobacteriaceae were responsible for all febrile episodes. Changing this protocol required time and effort, but the AMS program—which included leveraging local microbiological data, real-time reporting of bloodstream infection isolates with genotypic profiles, pharmacy alerts for prescriptions of restricted antimicrobials, prospective audits with 2 evaluations, immediate prescriber feedback, and regular staff meetings—effectively improved empirical and definitive antibiotic prescriptions. Similar programs in industrialized countries have demonstrated the benefits of evaluating antimicrobial consumption, accurately identifying pathogens, and implementing interventions by infectious disease specialists [10–13].

Reducing antimicrobial consumption was a central goal of our AMS program. Tailored initiatives to monitor vancomycin and meropenem prescriptions proved to be successful. By meticulously reviewing each indication, including dosing regimen and duration, we achieved significant reductions in unnecessary antimicrobial consumption. This approach has also been efficacious in other institutions [14].

Addressing the autonomy of treating physicians who prescribe antibiotics was another considerable challenge. Enhanced sharing of feedback with institutional leaders, which was based on prospective audits and discussions about clinical outcomes alongside stable hospital mortality rates, encouraged acceptance of the program [15]. Other significant challenges included improper antimicrobial administration, either alone or in combination with surgical prophylaxis, along with incorrect prescription timing, as observed in cancer centers in industrialized countries [16]. To address this issue, we formulated guidelines for surgical prophylaxis and enabled anesthesiologists to ensure the correct timing and administration of antimicrobials during surgery.

The limitations of our study include it being a single-center evaluation over a relatively short period, which restricts the generalizability of the findings to other centers in LMICs. In addition, we used the defined metric of DDD per 100 pd, which, although recommended by the WHO, is not commonly used in different settings. Furthermore, the intensive educational measures applied to health care workers may not be feasible in all centers.

In summary, our oncology center successfully implemented an AMS program, demonstrating that similar steps can be taken in LMICs to effectively curb the misuse of antimicrobials in hospital settings. Political commitment to improvement, leadership of infectious disease specialists in auditing prescriptions, multidisciplinary teamwork (among pharmacists, microbiologists, nurses, medical practitioners, and department heads), monitoring antibiotic consumption metrics, establishing guidelines for treating common infectious diseases, continuously educating health care professionals about antimicrobial treatment, and sharing of results and periodic feedback among members of other disciplines are among the most crucial steps that hospitals in LMIC can take in initiating an AMS program.

## Supplementary Material

ofae402_Supplementary_Data

## Supplementary Data

Supplementary materials are available at *Open Forum Infectious Diseases* online. Consisting of data provided by the authors to benefit the reader, the posted materials are not copyedited and are the sole responsibility of the authors, so questions or comments should be addressed to the corresponding author.

## Notes

***Acknowledgments*.** We sincerely thank our patients, whose understanding of the necessity of implementing an AMS program has greatly improved their quality of care. We also thank our institution's leaders for their steady support.

***Financial support*.** The core funds of Auna Peru sponsored this study.

***Patient consent statement*.** This study does not require patient consent.
