# Supplementary material for: Implementing an Antimicrobial Stewardship Program in an Oncology Center in Lima, Peru: A Model for Low- and Middle-Income Countries
Source: Open Forum Infect Dis. 2024 Jul 12;11(8):ofae402. doi: 10.1093/ofid/ofae402 (PMC11291919; doi:10.1093/ofid/ofae402)
Supplement: ofae402_Supplementary_Data [file ofae402_supplementary_data.docx]

1. Susceptibility pattern of the three most common gram-negative pathogens isolated from blood cultures*

*Escherichia coli*

|  | 2015 | 2016 | 2017 | 2018 | 2019 | 2020 | 2021 | 2022 | 2023 |
| --- | --- | --- | --- | --- | --- | --- | --- | --- | --- |
| Number of isolates | 100 | 80 | 75 | 80 | 79 | 60 | 100 | 166 | 117 |
| Ciprofloxacin | 17 | 0 | 23 | 25 | 32 | 46 | 31 | 10 | 25 |
| Amikacin | 83 | 100 | 100 | 100 | 100 | 100 | 100 | 100 | 93 |
| Cefttazidime | 20 | 48 | 46 | 50 | 53 | 48 | 70 | 51 | 57 |
| Piperacilin-tazobactam | 43 | 52 | 78 | 70 | 59 | 60 | 60 | 97 | 91 |
| Meropenem | 100 | 100 | 100 | 100 | 100 | 100 | 100 | 100 | 100 |
| Ertapenem | 100 | 100 | 100 | 100 | 100 | 100 | 100 | 100 | 100 |
| % of ESBL | 76 | 49 | 52 | 50 | 47 | 48 | 58 | 64 | 49 |
| % of CRE | 0 | 0 | 0 | 0.1 | 0 | 0 | 0 | 0 | 0 |

*Klebsiella pneumoniae*

|  | 2015 | 2016 | 2017 | 2018 | 2019 | 2020 | 2021 | 2022 | 2023 |
| --- | --- | --- | --- | --- | --- | --- | --- | --- | --- |
| Number of isolates | 50 | 53 | 36 | 30 | 40 | 28 | 10 | 39 | 57 |
| Ciprofloxacin | 39 | 67 | 43 | 50 | 59 | 70 | 70 | 69 | 50 |
| Amikacin | 82 | 100 | 100 | 100 | 98 | 100 | 100 | 100 | 86 |
| Cefttazidime | 25 | 46 | 43 | 57 | 42 | 47 | 50 | 79 | 62 |
| Piperaciline-tazobactam | 39 | 67 | 62 | 70 | 63 | 60 | 60 | 78 | 84 |
| Meropenem | 100 | 100 | 100 | 100 | 100 | 100 | 100 | 100 | 100 |
| Ertapenem | 100 | 100 | 100 | 100 | 100 | 100 | 100 | 100 | 100 |
| % of ESBL^&^ | 75 | 67 | 57 | 57 | 45 | 50 | 60 | 79 | 58 |
| % of CRE^~^ | 0 | 0 | 0 | 0 | 0 | 0 | 0 | 0 | 0 |

*Pseudomonas aeruginosa*

|  | 2015 | 2016 | 2017 | 2018 | 2019 | 2020 | 2021 | 2022 | 2023 |
| --- | --- | --- | --- | --- | --- | --- | --- | --- | --- |
| Number of isolates | 10 | 12 | 17 | 10 | 18 | 27 | 10 | 16 | 11 |
| Meropenem | 100 | 100 | 100 | 100 | 100 | 100 | 100 | 81 | 100 |
| Cefttazidime | 80 | 80 | 82 | 80 | 80 | 88 | 90 | 94 | 82 |
| Ciprofloxacin | 62 | 80 | 80 | 80 | 69 | 82 | 80 | 100 | 100 |
| Amikacin | 62 | 100 | 100 | 100 | 88 | 90 | 100 | 100 | 100 |
| Pip-tazo | 82 | 100 | 100 | 90 | 90 | 82 | 80 | 88 | 100 |
| Ceftoloxane-taz | NA | NA | NA | NA | NA | NA | NA | 100 | 100 |

1. Susceptibility pattern of *Staphylococcus aureus* isolated from blood cultures*

|  | 2015 | 2016 | 2017 | 2018 | 2019 | 2020 | 2021 | 2022 | 2023 |
| --- | --- | --- | --- | --- | --- | --- | --- | --- | --- |
| Number of isolates | 14 | 10 | 5 | 10 | 8 | 6 | 4 | 22 | 13 |
| % of MSSA^^^ | 100 | 100 | 100 | 100 | 100 | 100 | 100 | 100 | 100 |
| % MRSA^#^ | 0 | 0 | 0 | 0 | 0 | 0 | 0 | 0 | 0 |

*Values are % of susceptible strains unless noted otherwise

& ESBL: extended spectrum beta lactamase

~ CRE: carbapenem-resistant Enterobacteriaceae

^ MSSA: methicillin susceptible *S. aureus*

# MRSA: methicillin-resistant *S. aureus*
